# Risk factors for development of left ventricular thrombus after first acute anterior myocardial infarction-association with anticardiolipin antibodies

**DOI:** 10.1186/1477-9560-8-15

**Published:** 2010-09-19

**Authors:** Ertuğrul Okuyan, Barış Okcun, Mustafa H Dinçkal, Haşim Mutlu

**Affiliations:** 1 Istanbul University, Institute of Cardiology, Istanbul, Turkey; 2 Bagcilar Education and Research Hospital Cardiology Clinic, Istanbul, Turkey

## Abstract

**Background:**

Left ventricular thrombus(LVT] formation is a frequent complication in patients with acute anterior myocardial infarction(MI). LVT is associated with increased risk of embolism and higher mortality rates after acute MI. Anticardiolipin antibodies (ACA) are immunoglobulins that react with phospholipid-binding proteins interfering with the prothrombin activator complex. The effects of phospholipids on pathophysiology of cardiovascular thrombotic events are well known. In this study, we aimed to evaluate the importance of clinical and biochemical parameters including anticardiolipin antibodies on left ventricular thrombus formation after acute anterior MI.

**Methods and Results:**

Seventy patients with a first anterior AMI were prospectively and consecutively enrolled. Patients with previous MI, autoimmune disease, collagen vascular disease and arterial or venous thrombosis history were excluded from this study. At the time of hospitalization, key demographic and clinical characteristics were collected including age, gender, ethanol intake and presence of traditional risk factors for atherosclerosis (hypertension, diabetes, smoking, hyperlipidemia, positive family history). Patients were evaluated for echocardiographic data, blood chemistry and ACA. Two-dimensional and Doppler echocardiographic examinations were performed in all patients within the first week and at 14 days after MI. LV thrombus was detected in 30 (42.8%) patients. ACA IgM levels were significantly higher in the patient group with LV thrombus than in the group without thrombus (12.44 ±4.12 vs. 7.69 ± 4.25 mpl, p = 0,01). ACA IgG levels were also found higher in the group with LV thrombus (24.2 ± 7.5 vs.17.98 ± 6.45 gpl, p = 0.02). Multivariate analyses revealed diabetes mellitus, higher WMSI, lower MDT and higher ACA IgM and higher ACA IgG levels as independent predictors of left ventricular thrombus formation.

**Conclusions:**

Our data demonstrate that beside the low ejection fraction, lower MDT and higher wall motion score index, modestly elevated ACA IgM and ACA IgG levels are associated with LV thrombus formation in patients with anterior MI.

## Introduction

Left ventricular thrombus(LVT) formation is a frequent complication in patients with acute anterior myocardial infarction(MI). Left ventricular thrombus is associated with increased risk of embolism. Higher mortality rates have been reported in patients with LVT after acute MI, especially when these develop within the first 48 hours after infarction[1,2]. Although great majority of patients with LVT have large anterior infarcts with depressed global left ventricular systolic function, this is not the rule. Thrombi can also be found in some small apical infarcts with good global left ventricular systolic function and rarely in some inferior infarcts[3]. These facts indicate the complex nature of LVT formation. Factors other than infarct size and site may play role in development of LVT. Doppler derived mitral E wave deceleration time (MDT) has been found to be associated with LVT in some studies[4].

Anticardiolipin antibodies (ACA) are immunoglobulins that react with phospholipid-binding proteins interfering with the prothrombin activator complex. The effects of phospholipids on pathophysiology of venous and cardiovascular thrombotic events are well known. ACA levels have been found to be higher in young patients with coronary artery disease and proposed to be a risk factor in some studies[5]. ACA levels have also been found higher in patients with acute MI[5-8]. The question of whether anticardiolipin antibodies can be induced in response to tissue necrosis that occurs in myocardial infarction is unknown. There are some reports of patients with LVT and high ACA titers without MI and with normal left ventricular systolic function[9-11]. There is no detailed study evaluating the effects of ACA IgM and ACA IgG levels on development of left ventricular thrombus formation in patients with acute anterior myocardial infarction.

As LVT formation has a complex nature, we aimed to evaluate the factors associated with LVT formation in a group of patients with their first anterior wall MI who recieved different kinds of therapies. We also aimed to evaluate the importance of anticardiolipin antibodies on LVT formation after acute anterior wall MI in a population who did not present features of antiphospholipid syndrome(APS). By selecting this population, we aimed to evaluate if modest increase in these antibody levels associate with LVT formation or not. We also tried to determine if ACA levels are higher after an acute event(MI).

## Methods

Seventy patients with a first anterior AMI were prospectively and consecutively enrolled. Patients were required to meet the following criteria: (1) age <70 years, (2) chest pain lasting >30 minutes, (3) more than 2 mm ST segment elevation at least in 2 consecutive anterior precordial leads of the electrocardiogram(ECG),(4) initial echocardiogram performed within 48 hours following admission.

Patients with previous MI, autoimmune disease, collagen vascular disease, arterial or venous thrombosis history,thrombotic hematological disorders, history of heart failure were excluded from the study. By excluding these subjects, we aimed to compose a homogenous patient group to avoid misinterpretation caused by results of patients with those pathologies. In addition, patients with atrial fibrillation, aortic stenosis, renal dysfunction(history of renal failure or serum creatine level ≥1.3 mg/dl), hepatic dysfunction(history of hepatic disease or serum transaminase level elevations greater than 2 times the upper limit of normal), patients with permanent pacemakers and patients with low quality echocardigraphic images were also excluded from the study.

At the time of hospitalization, key demographic and clinical characteristics were collected including age, gender, ethanol intake and presence of traditional risk factors for atherosclerosis (hypertension, diabetes, smoking, hyperlipidemia, positive family history). A complete diagnostic evaluation was performed for each patient; this included blood chemistry, blood cell counts, erythrocyte sedimentation rate, fibrinogen levels, ACA IgM and ACA IgG levels, coagulation panel, ECG and chest × ray.

### Therapy

Thrombolytic therapy either t-PA(accelerated t-PA protocol;15 mg intravenous bolus, followed by 0.75 mg/kg infused over the next 60 minutes) or streptokinase(1500000 unit infused over 60 minutes) were administered to 40 of 70 patients(26 streptokinase, 14 t-PA). Primary percutaneous coronary intervention(PCI) with stenting was performed to 5 patients. After prompt clinical and laboratory evaluation, reperfusion therapy was not given to other patients due to late admission. Acetyl Salicylic Acid(ASA) (160-300 mg), heparin, beta blockers and angiotensin converting enzyme(ACE) inhibitors were given to all patients. Statins, nitrates and positive inotropic agents were administrated if neccessary. Warfarin was subsequently administered if LVT was present on echocardiography and there was no contraindication for anticoagulant therapy. Patients were followed at outpatient clinic on a monthly basis after hospital discharge.

### Echocardiography

Echocardiography was performed within 48 hours of admission with an Acusone 128 XP/5 ultrasound system with 3.5 MHz transducer(Mountain view, CA. USA.) The procedure was repeated on day 4, day7 and day 14 of hospitalization. The images were recorded and analyzed by 2 independent cardiologist, who were blinded to each others interpretation. The diagnosis of left ventricular thrombus was made when an echodense mass was visible throughout the cardiac cycle in at least two echocardiographic views with associated asynergy(akinesis or dyskinesis) of the adjacent myocardium[12,13] and with a distinct magrin from the left ventricular wall detected within the left ventricular cavity. And if both of the cardiologists were completely agree about the presence of thrombus.

The Wall Motion Score Index (WMSI) was obtained semiquantitatively on initial echocardiographic examination by dividing the left ventricule to 16 segments as proposed by the American Society of Echocardiography[14]. Segmental wall motion was graded as follows: normal motion at rest(score = 1); hypokinetic-marked reduction in endocardial motion and systolic thickening(score = 2); akinetic-virtual absence of inward motion and systolic thickening(score = 3); and dyskinetic-paradoxical wall motion away from the centre of the left ventricle in systole(score = 4)[15]. The WMSI was calculated by summation of individual segment scores divided by the number of interpreted segments. Left ventricular ejection fraction was estimated using Simpson's modified biplane method[16].

Left ventricular diastolic filling patterns were determined by the mitral inflow pulsed-wave Doppler examination with a 2.5-MHz transducer. In the apical 4-chamber view, the Doppler sample volume was placed in the middle of the LV inflow tract 1 cm below the plane of mitral annulus between the mitral leaflet tips, where maximal flow velocity in early diastole was recorded. Special care was taken to align the sample volume as close to perpendicular as possible to the mitral annular plane. From Doppler spectra of 3 to 5 consecutive cardiac cycles, average values were calculated for the following transmitral parameters: peak early(E) and late(A) transmitral filling velocities, their ratio(E/A), and the deceleration time of E wave velocity(MDT).

### Measurement of Antibodies

All the tests were done in duplicate with sera stored at -70°C, and mean value was obtained. Determination of ACL antibodies was performed by standarized ELISA for both IgG and IgM isotypes as previously described[17]. Results are expressed as GPL units for IgG and MPL units for IgM ACL antibodies with 1 GPL or MPL unit being equivalent to 1 μg/ml of an affinity-purified Standard[18]. All the tests met the guality control standards as determined by the manufacturer. Referrence ranges of ACA assay in our study for ACA IgM was <9.8 MPLU/ml and <13.3 MPLU/ml for IgG. Values above these cut-off points were considered as elevated levels in this study.

### Coronary Angiography

Coronary angiography was performed to 46 of 70 patients during hospitalization. The angiographic findings were graded according to the American Heart Association (AHA) classification[19], in which significant coronary stenosis is defined as ≥75% narrowing of the major epicardial artery.

### Statistical Analyses

Data were presented as mean ± standard deviation. The Chi-square test was used for the comparison of non-parametric data and T-test was used to compare continuous variables. The Cox regression model was used to identify variables associated with thrombus formation(univariate analysis). Step-wise multiple regression analysis was performed to determine the correlation between left ventricular thrombus formation and each independent variable

P value of <0.05 with 95% confidence interval was considered statistically significant. All tests were performed with SPSS(Statistical Package for Social Sciences, Chicago, IL,USA) for Windows 10.0.

The study complies with the Declaration of Helsinki and the Ethics Committee of local university faculty of medicine has approved the research protocol. Informed consent has been obtained from all included subjects.

## Results

LVT was detected in 30 of 70 patients(42.8%). Patients were divided into two groups according to the presence of LVT detected by echocardiography(Group 1 with LVT, Group 2 without LVT). The cumulative percentage of identified thrombus in each echocardiographic examination was 16.66%(5) within 48 hours; 50%(15)within 96 hours; 80%(24) at 1 week and 100%(30) at 2 weeks.

There were no significant differences between two groups in terms of age, gender, hyperlipidemia, smoking, family history of coronary artery disease and hospitalization duration. Baseline characteristics of patients were shown on Table 1. Previous history of hypertension was significantly higher in the patient group without thrombus(p = 0.02), whereas history of diabetes mellitus was significantly lower in this group(p = 0.04).

**Table 1:** Baseline characteristics of patients

	Without thrombi	With thrombi	p value
Number of patients	40(%56.2)	30(%42.8)	NS

Age(y), mean ± SD	52 ± 12	56 ± 15	NS

Female (%)	4(%11.76)	2(%6.66)	NS

Male (%)	30(%88.23)	28(%93.33)	NS

Hypertension(%)	14(%41)	5(%16)	= 0,02

Hyperlipidemia(%)	20(%58)	20(%66)	NS

Diabetes (%)	1(%2.9)	15(%23)	= 0,04

Smoking(%)	27(%79)	26(%86)	NS

Hospitalization duration	7 ± 2	8 ± 2	NS

KILLIP class at admision	1.47 ± 0.92	2.31 ± 1	= 0.04

NS = Non significant.

Peak CPK levels were significantly higher in patient group with left ventricular thrombus than without thrombus (4170 ± 1250.60 U/L, 2580.24 ± 1468.25 U/L, respectively p < 0.001, Table 2).

**Table 2:** Biochemical and echocardiographic findings between two groups.

	Without thrombi	With thrombi	p value
CPK(peak)	2580.24 ± 1468.25 U/L	4170 ± 1250.60 U/L	< 0.001

WMSI(day 1)	1.81 ± 0.25	2.17 ± 0,21	< 0.001

EF(%,day 1)	41 ± 3.7	30 ± 5.3	= 0.001

MDT	155.26 ± 44.61 ms	127.34 ± 39.07 ms	= 0.007

ACA IgM	7.69 ± 4.25 mpl	12.44 ± 4.12 mpl	= 0.01

ACA IgG	17.98 ± 6.45 gpl	24.2 ± 7.5 gpl	= 0.02

WMSI = Wall motion score index; EF = Ejection fraction; CPK = Creatine phospho kinase; MDT = Mitral E wave Deceleration Time; ACA=Anticardiolipin antibody; NS = Non significant.

Mean left ventricular WMSI of patients with left ventricular thrombus was significantly higher than patients without thrombus (2.17 ± 0,21 and 1.81 ± 0.25 respectively, p < 0.001, Table 2). Initial (Day 1) ejection fractions (EF) of patient group with left ventricular thrombus was also significantly lower than patient group without thrombus (30 ± 5.3%and 41 ± 3.7% respectively, p = 0.001, Table 2). Doppler-derived mitral E wave deceleration time(MDT) was significantly lower in patients with LVT than without LVT(127.34 ± 39.07 ms versus 155.26 ± 44.61 ms respectively, p = 0.007).

ACA IgM levels were significantly higher in the patient group with LV thrombus than in the group without thrombus (12.44 ± 4.12 vs. 7.69 ± 4.25 mpl, p = 0,01). ACA IgG levels were also found higher in the group with LV thrombus (24.2 ± 7.5 vs.17.98 ± 6.45 gpl, p = 0.02).

Thrombolytic therapy has been administrated to 40 patients. LVT was observed in 16 of them. Reperfusion therapy was not given to 25 patients, and LVT was observed in 12 of them. Five patients have undergone primary PCI and in 2 of them LVT was developed. These findings were statistically nonsignificant.

46 patients have undergone diagnostic coronary angiography(66%). Significant coronary stenosis was defined as ≥75% narrowing of a major epicardial artery. According to this criteria 19(41%) of patients had single vessel disease, 18(39%) had 2-vessel disease and 8(17%) had 3-vessel disease and one patient had completely normal coronary arteries. LVT was observed in this patient with normal coronary arteries. LVT was observed in 7 of 19(37%, p = nonsignificant) patients with single vessel disease, 9 of 18(50%, p = nonsignificant) patients with 2-vessel disease and 7 of 8(87.5%, p < 0.001) patients with 3-vessel disease.

Univariate analysis(Table 3) showed that LV thrombus formation was associated with a higher ACA IgM levels (beta = 0.02, p = 0.01), a higher ACA IgG levels (beta = 1.75, p = 0.02), a lower initial ejection fraction (beta = 0.04, p = 0,001), lower MDT(beta = 0.03, p = 0.02) and a higher initial wall motion score index (beta = -0.86, p = 0.01).

**Table 3:** Predictors of thrombus formation according to univariate analysis

	Beta	P value
Age	0.001	NS

Gender(F/M)	0.002	NS

Hypertension	-0.84	0,02

Hyperlipidemia	1.4	NS

Diabetes	0.9	0,04

Smoking	1.34	NS

WMSI(day 1)	-0.86	= 0.01

EF(day 1)	0.04	0.001

CPK(peak)	1.75	< 0.001

ACA IgM	0.02	= 0.01

ACA IgG	1.75	= 0.02

MDT	0.03	= 0.02

WMSI = Wall motion score index; EF = Ejection fraction; CPK = Creatine phospho kinase; ACA = Anticardiolipin; MDT = Mitral E wave deceleration time; NS = Non significant.

In multivariate analyses(Table 4), diabetes mellitus(odds ratio, 1.26; 95% confidence interval, 1.24 to 5.29; p = 0.03), higher WMSI(odds ratio, 1.95; 95% confidence interval 1.33 to 6.57; p = 0.001), lower MDT(odds ratio, 1.67; 95% confidence interval 1.49 to 7.53; p = 0.001) and higher ACA IgM (odds ratio, 1.27; 95% confidence interval, 1.23 to 4.19; p = 0.04) and higher ACA IgG levels(odds ratio, 1.41: 95% confidence interval, 1.34 to 4.42 p = 0.03) were independent predictors of left ventricular thrombus formation.

**Table 4:** Multiple regression analysis for LVT formation and related parameters

Independent variables	OR	p value
Diabetes Mellitus	1.26	0.03

WMSI	1.95	0.001

MDT	1.67	0.001

ACA IgM	1.27	0.04

ACA IgG	1.41	0.03

Four embolic cerebrovascular accidents developed in the group with LVT during the 6-month follow-up period eventhough they were under ASA and warfarin therapy. Two of them died. Ischeamic cerebrovascular event was not observed in patient group without thrombus. Six-month mortality of patients was 16.66%(5/30) with LVT, and 5%(2/40) without LVT(P < 0.001).

## Discussion

Left ventricular thrombosis is one of the most often and devastating complication of acute myocardial infarction. In different studies, incidence of left ventricular thrombosis was found to be between 28% and 54%[1,12,20-22]. In our study, the incidence of left ventricular thrombi was found to be 42.85%. This value seems to be higher than expected. Currently preferred reperfusion strategy for acute MI is PCI. However only a minority of patients was treated with PCI and a significant proportion recieved no PCI neither thrombolysis. Therefore subjects in this study are not representative for contemporary treatment of acute anterior MI in respect to PCI era. But it should be noted that, PCI availability is still limited in many centers worldwide, especially in developing countries and pain to needle time is still extended in many areas. Our study population consists of a significant number of patients referred from other centers and other cities. For that reason many patients could not recieve reperfusion therapy due to late admission. This might be the one of the leading causes of high incidence of LVT in our study.

Higher mortality has been reported in patients with left ventricular thrombi after infarction, especially when these develop within the first 48 hours after infarction[1,2]. Left ventricular thrombosis is associated with increased embolism risk [12,23,24]. Therefore, better understanding of the circumstances in which left ventricular thrombosis occurs may influence patient management. As mentioned before, in our study four embolic cerebrovascular accidents developed in the group with LVT during the 6-month follow-up period though they were under ASA and warfarin therapy. Two of them died. Any cerebrovascular event was not observed in patient group without thrombus. Six-month mortality of patients was 16.66%(5/30) with LVT, and 5%(2/40) without LVT(P < 0.001).

Prospective echocardiographic studies investigating the effect of thrombolytic therapy on thrombus formation after myocardial infarction revealed controversial results. Some of them did not demonstrate a reduction of LVT incidence after thrombolytic therapy[25], while some other studies showed that thrombolytic therapy, by preserving the left ventricular function, reduced LVT incidence[26-29]. In our study, thrombolytic therapy(either tPa or streptokinase) has administrated to 40 patients. LVT was observed in 16 of them(40%). Thrombolytic therapy was not given to 25 patients, and LVT was observed in 12 of them (48%). Of 5 patients undergoing primary PCI, 2 developed LVT(40%). Although thrombolytic therapy seems to reduce LVT incidence in our study, it is not statistically significant. In the modern era, there is widespread use of reperfusion therapies such as thrombolysis and primary PCI for the management of AMI. In recent years, primary PCI with stenting has become the reperfusion therapy of choice for AMI in institutions with emergency cardiac catheterization facilities. Primary PCI achieves full and sustained reperfusion, which promotes early recovery of the infarcted myocardium and may therefore theoretically decrease the incidence of LV thrombosis. Karla et al. in their retrospective study found that, the incidence of LVT among anterior MI patients treated with primary PCI was 10% [30]. In a recent study from Rabbani et al. incidence of LVT remains high despite PCI for acute anterior wall MI(35%) [31].

In our study, multivariate analysis showed that; diabetes mellitus, higher WMSI, lower MDT, higher ACA IgM and IgG levels were independent predictors of LVT formation. In many studies, it is noted that higher WMSI is associated with worse prognosis and high risk of LVT formation[4,13,32,33]. WMSI shows severity of left ventricular systolic dysfunction, so it is reasonable to see high incidence of LVT in patients with higher WMSI. A MDT of >130 ms was classified as nonrestrictive, and < 130 ms was defined as restrictive. This cut off point has been shown to be consistent with restrictive hemodynamics and a powerfull independent predictor of unfavorable outcome after acute myocardial infarction[33,34]. The size of infarct zone has been shown to influence the diastolic filling pattern, with large infarcts exhibiting a restrictive filling pattern[35,36]. Therefore a short MDT, indicative of a restrictive filling pattern, might simply reflect an increasing infarct size and consequently a higher risk of left ventricular dilatation.WMSI and MDT can be simply determined by 2-D and Doppler echocardiographic examinations and if performed earlier after acute myocardial infarction, they can help to identify high risk patients for LVT development.

The association of anticardiolipin antibodies with acute myocardial infarction has been shown in several studies[37-40]. Our study is unigue among others because it reveals association between anticardiolipin antibodies and left ventricular thrombus formation for the first time. Also some of the mentioned studies reveal direct association of IgG type anticardiolipin antibodies with acute myocardial infarction and inverse association of IgM type anticardiolipin andibody levels with reccurent cardiac events. But in our study, both IgM and IgG type of antibodies seem to be associated with left ventricular thrombus formation. Although we find a statistical correlation between high IgG and high IgM ACA levels and occurance of LVT, the actual values of IgG and IgM ACA are not especially high. Thus the mean levels of IgG ACA are about 20 GPLU and of IgM ACA about 10 MPLU. In patients who have antiphospholipid syndrome itself the levels are frequently far higher. The combination of these modest antibody levels and exclusion of patients in whom these ACA antibodies are likely to be pathogenic(those with autoimmune disease, arterial or venous thrombosis, collagen vascular disease etc.) means that we are looking at importance of low-level non-pathogenic ACA.

According to the current revised laboratory criteria for APS[41], both lupus anticoagulant and aCL IgG and IgM are maintained as laboratory APS criteria, and IgM and IgG anti-ß2 glycoprotein-I assays are added in the revised criteria. Medium and high titers of IgG and IgM aCL antibodies associate with clinical manifestations of APS, and were selected as criteria in Sapparo[42] during formulation of the international preliminary classification criteria for APS. However, the threshold used to distinguish moderate-high levels from low levels has no Standard[43], and definition of the level that best corresponds to the risk of clinical manifestations is difficult[44]. Based on the best available evidence, and until an international consensus is reached, the committee introduced a clear statement on threshold for positive: > 40 GPL or MPL units. Referrence ranges of ACA assay in our study for ACA IgM was <9.8 MPLU/ml and <13.3 MPLU/ml for IgG. Values above these cut-off points were considered as elevated levels in this study. ACA IgM and IgG levels were above these cut-off points for 34 and 45 patients respectively.

The question of whether anticardiolipin antibodies can be induced in response to tissue necrosis that occurs in myocardial infarction is unknown[45]. However, there is clinical evidence that anticardiolipin antibodies precede the development of first MI and that anticardiolipin titers are stable for up to 3 months after MI[37,38]. These studies indicate that anticardiolipin antibodies are not generated by tissue necrosis but rather they participate in the pathogenesis of MI. The relatively higher levels of ACA in our study population can not answer the question of whether anticardiolipin antibodies can be induced in response to tissue necrosis that occurs in myocardial infarction.

In a meta-analysis by Gali et al.[46] Lupus anticoagulant was found to have the strongest association with thrombosis, whereas only medium and high titers of ACA IgG were linked to thrombosis. In our study, even modest increase in the levels of both ACA IgG and IgM were associated with left ventricular thrombosis. Prospective studies of large populations needed to explore this association further. Numerous mechanisms have been proposed to explain thrombus formation by antiphospholipid antibodies, such as direct endothelial damage, enhanced platelet aggregation, and inhibition of endogenous anticoagulants. These antibodies, in the presence of other hemostatic defects and impaired LV function, may affect surface factors and promote clot formation.

Diagnostic coronary angiography was performed to 46 of 70 patients. There is a linear association between number of diseased coronary artery and the LVT formation in our study. It is reasonable to see more severe LV systolic dysfunction with multivessel coronary artery disease and thus LVT formation.

In summary, our data demonstrate that beside the low ejection fraction, lower MDT and higher wall motion score index, even modestly increased levels of ACA IgM and ACA IgG are associated with LV thrombus formation in patients with anterior MI. Prospective, larger scale studies will be a better guide for establishing the impact of ACA on LVT formation.

## Limitations

There are many limitations of this study. At first, it is observational and therefore allows us to explore associations. It cannot provide casual evidences, but only states hypotheses for further research. Moreover, the number of participants is relatively small.

As stated in the text, the subjects in this study are not representative for contemporary treatment of acute anterior MI. A significant proportion of patients admitted very late in the course of their MI and during study period there were no enough arrangements for primary PCI after office hours.

Colinearity between the diffent types of anticardiolipin antibodies might have affect the results at multivariate analysis. We did not repeat the ACA assays after a certain period in this study.

Several conceivable risk factors were found at multivariate analysis: diabetes mellitus, higher WMSI and lower MDT. In this respect, an increase in mean level of IgG and IgM aCL may appear as irrelevant findings in relation to the poor standardization of the assay, its possible unreliable results close to acute events. But, it must be stated that the main aim of this study is to document all conceivable risk factors associated with LVT formation.

## Competing interests

The authors declare that they have no competing interests.

## Authors' contributions

EO enrolled the eligible patients and he was directly responsible for collecting data and follow-up of patients. BO and HM performed the echocardiographic examinations and checked the accuracy of obtained data. HM participated in design and coordination of study. MHD performed the statistical analysis. All authors read and approved the final manuscript.
